# Biomarker identification through spatial proteomics for the characterization of indeterminate thyroid nodules

**DOI:** 10.1007/s12020-025-04383-9

**Published:** 2025-08-11

**Authors:** Giulia Capitoli, Antonio Maria Alviano, Nicole Monza, Lisa Pagani, Isabella Piga, Davide Paolo Bernasconi, Angela Greco, Davide Leni, Alice Maggioni, Andrea-Valer Gatti, Fausto Maffini, Nicola Fusco, Mattia Garancini, Fulvio Magni, Stefania Galimberti, Fabio Pagni, Vincenzo L’Imperio, Vanna Denti

**Affiliations:** 1 Bicocca Bioinformatics Biostatistics and Bioimaging Research Centre - B4, Department of Medicine and Surgery, University of Milano-Bicocca, Monza, Italy; 2 Biostatistics and Clinical Epidemiology, Fondazione IRCCS San Gerardo dei Tintori, Monza, Italy; 3 Pathology, Fondazione IRCCS San Gerardo dei Tintori and Center of Digital Medicine, Department of Medicine and Surgery, University of Milano-Bicocca, Monza, Italy; 4 Proteomics and Metabolomics Unit, Department of Medicine and Surgery, University of Milano-Bicocca, Monza, Italy; 5 Department of Radiology, Fondazione IRCCS San Gerardo dei Tintori, Monza, Italy; 6 Endocrine and Metabolic Surgery Unit, Department of Surgery, IRCCS Fondazione San Gerardo dei Tintori, Monza, Italy; 7 Division of Pathology, European Institute of Oncology IRCCS, Milan, Italy; 8 Department of Oncology & Hemato-Oncology, University of Milan, Milan, Italy; 9 Present Address: Independent Researcher, Cagliari, Italy

**Keywords:** Thyroid cancer, Fine-needle aspiration, Cytology, Digital pathology, Computational pathology, MALDI-MSI

## Abstract

**Purpose:**

The identification of novel molecular biomarkers may assist in the characterization of indeterminate thyroid nodules, which pose significant diagnostic challenges. Here, we aimed to explore the potential of proteomic analyses to support biomarker discovery in challenging thyroid lesions.

**Methods:**

Linear Discriminant Analysis (LDA) was applied to Matrix-Assisted Laser Desorption Ionization Mass Spectrometry Imaging (MALDI-MSI) data from 44 thyroid neoplasms to select the most impactful molecular features for the classification of different tumor histologies, as well as for the distinction between NRAS-mutant (mNRAS) and NRAS-wild-type (wtNRAS) tumors. Relevant peaks were subsequently identified through nanoscale liquid chromatography electrospray ionization tandem mass spectrometry (nLC–ESI-MS/MS).

**Results:**

The LDA selected nine relevant molecular markers distinguishing noninvasive follicular thyroid neoplasms with papillary-like nuclear features (NIFTPs) from other tumor histologies (balanced accuracy = 73%), as well as 19 relevant markers able to identify mNRAS cases (balanced accuracy = 84%). Nine differentially expressed proteins were putatively identified: among them, ATP-dependent RNA helicase DDX42 showed a similar distribution between NIFTPs and papillary thyroid carcinomas (PTCs) / follicular variant PTCs (FVPTCs), while the distribution of the Histone H4 signal was similar between NIFTPs and follicular adenomas (FAs). In addition, Protein disulfide-isomerase A1 and Complement C4-B were overexpressed in wtNRAS compared to mNRAS cases, regardless of histology.

**Conclusion:**

The LDA-selected features enable to distinguish NIFTPs from morphologically similar lesions and to discriminate between mNRAS and wtNRAS cases. The identified markers might complement genetic analyses and provide insights into the distinct pathogenic drivers behind the development of mNRAS compared to wtNRAS lesions.

## Introduction

Given the high incidence of thyroid nodules and the associated risk of malignancy, an accurate characterization of such lesions is of paramount importance [1]. Despite being the gold standard for risk stratification purposes, fine-needle aspiration cytology (FNAC) yields indeterminate results in up to 30% of cases, many of which undergo diagnostic surgery [2]. To prevent this, various ancillary classifications based on radiology have been proposed [3, 4], possibly complemented by genetic analyses, with still perfectible discriminative capabilities for the different follicular-patterned lesions [5]. The recently updated histological terminologies (e.g. Noninvasive Follicular Thyroid Neoplasm with Papillary-like nuclear features, NIFTP) have added additional complexity to the diagnostic workup of borderline thyroid tumors, also considering their marked heterogeneity. In this context, the application of Matrix Assisted Laser Desorption Ionization Mass Spectrometry Imaging (MALDI-MSI) has shown promise in the characterization of the molecular fingerprints of different thyroid lesions [1, 5], demonstrating encouraging preliminary results even in the identification of NIFTP molecular signatures [6]. Moreover, statistical classification algorithms have emerged as a promising tool to support the diagnostic process of tumors [7], including in the field of thyroid cytology to distinguish some challenging entities (e.g. NIFTP) from more aggressive or indolent lesions [10, 11]. Here, we tested a MALDI-MSI-based approach for biomarker discovery on a challenging series of thyroid neoplasms to assess whether the classification ability of proteomics can be improved by statistical classification algorithms, with a specific focus on the distinction between NIFTPs and histologically similar neoplasms (e.g., follicular adenoma [FA], papillary thyroid carcinoma [PTC], and invasive encapsulated and infiltrative follicular variants of PTC [iE-FVPTC and I-FVPTC, respectively]). Within follicular-patterned tumors, we also evaluated the performance of this combined approach in the distinction between NRAS-mutant (mNRAS) and NRAS-wild-type (wtNRAS) lesions.

## Materials and methods

### Cases

The study cohort was composed of 44 patients who underwent thyroid surgery for different thyroid tumors at the IRCCS Fondazione San Gerardo dei Tintori, Monza, Italy. Two different areas were selected for each tumor sample from the original formalin-fixed paraffin-embedded (FFPE) tissue block by an experienced thyroid pathologist (FP) to build a tissue microarray (TMA) using the semi-automatic arrayer ISE Galileo TMA CK 4500 through the ISE Galileo TMA R4.30 software (Integrated Systems Engineering, Milan, Italy). The TMA was tested for the NRAS Q61R mutation by immunohistochemistry (rabbit monoclonal antibody, clone RST-NRAS, dilution 1:20) on DAKO Omnis (Agilent, Santa Clara, CA, USA).

For each case, demographic (age and sex), clinical (nodule laterality, nodule diameter), and cytological characteristics were extracted. Cytological diagnoses were reported according to the TIR classification of the Italian Society of Pathology (SIAPEC) [12]. Approval was obtained from the local ethics committee (FINAL-TIR PU 3581/21), and all subjects enrolled in the study signed an informed consent. The study was not part of a clinical trial.

### MALDI–MSI analysis

Sections from the obtained TMA block were treated as previously described for MALDI-MSI preparation [6]. The mass spectra were acquired in the reflectron positive mode, within the range of *m/z* 700 to 3000, using a rapifleX MALDI Tissuetyper (Bruker Daltonics, Bremen, Germany) MALDI-TOF/TOF MS equipped with a Smartbeam 3D laser operating at 2 kHz frequency. The MALDI-MSI images were acquired with a single-spot laser setting of 10 μm and a scan range of 6 × 6 μm. A mixture of standard peptides within the mass range of *m/z* 750 to 3150 (PepMix I, Bruker Daltonics) was used for the external calibration, directly applied on the glass slide (mass accuracy <10 ppm). FlexControl 4.0 (Bruker Daltonics) was used to set up the instrument parameters for the acquisition method, and FlexImaging 5.0 (Bruker Daltonics) for the MALDI–MSI analysis visualization. Next, the matrix was removed and the slides were stained with hematoxylin and eosin (H&E). Finally, the slides were converted to a digital format using a MIDI II digital scanner (3DHISTECH, Budapest, Hungary), allowing the integration of the proteomic and morphological data. Within each core, an expert thyroid pathologist (FP) annotated different regions of interest (ROIs), separating normal tissue (thyroid parenchyma and stroma) from lesional areas, as reported in Fig. S1, for a total of 144 ROIs of lesional areas. The annotated regions were then imported into SCiLS Lab 2024 Pro software (Bruker, Bremen, Germany) and the corresponding average mass spectrum was extracted from each ROI.

### nLC–ESI-MS/MS sample preparation and analysis

After the MALDI–MSI analysis, a nanoscale liquid chromatography electrospray ionization tandem mass spectrometry (nLC–ESI-MS/MS) analysis for peptide identification was performed, as previously described with some modifications [6]. The matrix collected from each slide was dried with a vacuum centrifugal evaporator (Hetovac, Savant) and resuspended in 50 µL of phase A (98:2:0.1; water/acetonitrile/trifluoroacetic acid). The nLC–ESI MS/MS analysis was performed using a Dionex UltiMate 3000 rapid separation (RS) LC nano system coupled with an Impact HD UHR-QqToF (Bruker Daltonics). Sample desalting and concentration were carried out using a pre-column (Dionex, Acclaim PepMap 100 C18, cartridge, 300 μm), and the peptides were separated with a 50 cm column (Dionex, ID 0.075 mm, Acclaim PepMap100, C18) at 40 °C, using a 240 min gradient from 96% to 2% of phase A (0.1% formic acid), whilst phase B was 0.08% formic acid:acetonitrile (80:20). The MS was operating in data-dependent acquisition mode. Compass DataAnalysis v4.1 software (Bruker Daltonics) was used to calibrate, deconvolute and convert the acquired raw data prior to the protein identification and quantification. The Peaks Studio X-Plus (Bioinformatics Solutions Inc., Waterloo, ON, CA) was used for protein identification. The parameters were set as follows: trypsin as the digestive enzyme, no fixed modifications, oxidation (M) and FFPE + 12 and FFPE + 30 as the variable modifications. The precursor mass error and the fragment mass error tolerances were set at 20 ppm and 0.05 Da, respectively. The identification engine used the Swissprot database, selecting the homo sapiens taxonomy. For identification, a peptide false discovery rate (FDR) of ≤1% was applied. The proteins were considered identified if they had at least one unique significant peptide (−10logP ≥20). The signals of interest present within the MALDI–MSI dataset were correlated with the positively identified peptide sequences obtained using the nLC–ESI-MS/MS. An identification was putatively assigned to a signal if an error of <100 ppm was noted between the two measured *m/z* values.

### Statistical analysis

The database containing the average spectra of each ROI underwent a pre-processing workflow. This process included baseline subtraction (TopHat algorithm), normalization (Total Ion Current algorithm), spatial denoising, alignment, and peak picking. As a result, 373 *m/z* molecular features indicating the molecular abundance contained in each of the 144 ROIs were detected. Linear Discriminant Analysis (LDA) was applied for feature selection and classification [13, 14]. LDA aims to reduce dimensionality by finding a lower-dimensional subspace that maximizes the separability of the samples [15], assuming that the features might be dependent. The optimal number of features was selected controlling for FDR. The usual performance metrics, i.e. sensitivity (Sn), specificity (Sp), positive and negative predictive values (PPV and NPV, respectively) were calculated on the entire cohort, considering only the selected features. Balanced accuracy was used instead of generic accuracy to account for differences in sample size between diagnostic classes. Features selected by LDA were compared for each task of the study: discriminating between tumor histotypes and differentiating between mNRAS and wtNRAS samples. Pairwise Wilcoxon-tests were used to quantify the differences between lesions and samples with and without positivity to NRAS Q61R, accompanied by the associated fold-change, defined as the ratio of the mean values between two classes. All the analyses were performed using R software version 4.2.2 (The R Foundation For Statistical Computing, Vienna, Austria) with the MixOmics library (http://mixomics.org/install/).

## Results

### Cases

The study cohort consisted of 44 cases, corresponding to 88 TMA cores (two cores were collected for each patient) and a total of 144 pathologist-annotated ROIs. The clinicopathological characteristics of enrolled subjects are summarized in Table 1. The majority of patients were female (n = 36, 82%), with a median age of 51 years (interquartile range [IQR] 45–63). The median diameter of the nodules was 1.25 cm (IQR 0.88–1.80), and most nodules were located in the right lobe (n = 19, 43.2%), with significantly larger nodules belonging to the FA category (median size = 3 cm, IQR 2.8–3.13, p = 0.002). The final histological diagnoses included 18 cases (44 ROIs) of conventional PTC, 7 (23 ROIs) of I-FVPTC, 3 (5 ROIs) of iE-FVPTC, 4 (8 ROIs) of FA, and 12 (34 ROIs) of NIFTP. Given the small number of ROIs within the I-FVPTC and iE-FVPTC classes, these PTC variants were grouped into a single category (i.e., FVPTC) for subsequent analyses. A higher prevalence of the NRAS Q61R mutation was observed in follicular-patterned lesions (50% of FAs, 28.6% of I-FVPTCs, and 50% of NIFTPs) compared to classic PTCs, where it was found in only one case (6.2%, p = 0.082).

**Table 1:** Clinical and pathological features of patients enrolled in the study

Characteristic	Overall, n = 44	FA, n = 4	I-FVPTC, n = 7	iE-FVPTC, n = 3	NIFTP, n = 12	PTC, n = 18	p-value^c^
Age (years)^a^	51 (45, 63)	45 (30, 60)	58.1 (50, 64)	52 (43, 64)	56 (48, 66)	48 (44, 50)	0.208
Sex^b^							0.464
F	36 (82)	4 (100)	4 (57.1)	3 (100)	10 (83)	15 (83)	
M	8 (18)	0 (0)	3 (42.9)	0 (0)	2 (17)	3 (17)	
NRAS mutational status^b^							0.082
MUT	11 (26.2)	2 (50)	2 (28.6)	0 (0)	6 (50)	1 (6.2)	
WT	31 (73.8)	2 (50)	5 (71.4)	3 (100)	6 (50)	15 (93.8)	
N/A	2	0	0	0	0	2	
Nodule laterality^b^							0.941
I	2 (4.5)	1 (25)	0 (0)	0 (0)	1 (8.3)	0 (0)	
L	15 (34.1)	1 (25)	3 (42.9)	1 (33.3)	4 (33.4)	6 (33.4)	
MF	8 (18.2)	0 (0)	1 (14.2)	1 (33.3)	2 (16.7)	4 (22.2)	
R	19 (43.2)	2 (50)	3 (42.9)	1 (33.3)	5 (41.6)	8 (44.4)	
Nodule size (cm)^a^	1.25 (0.88, 1.80)	3.00 (2.80, 3.13)	0.99 (0.60, 1.40)	0.60 (0.55, 0.70)	1.70 (0.98, 2.05)	1.15 (0.93, 1.50)	0.004
Cytological classification^b^							0.140
Tir1	1 (3.5)	0 (0)	0 (0)	0 (0)	1 (12.5)	0 (0)	
Tir2	8 (27.6)	1 (50)	1 (25)	0 (0)	4 (50)	2 (14.3)	
Tir3	4 (13.8)	1 (50)	0 (0)	1 (100)	2 (25)	0 (0)	
Tir4	7 (24.1)	0 (0)	1 (25)	0 (0)	1 (12.5)	5 (35.7)	
Tir5	9 (31)	0 (0)	2 (50)	0 (0)	0 (0)	7 (50)	
N/A	15	2	3	2	4	4	
Lymphovascular invasion^b^							0.055
Absent	37 (84.1)	4 (100)	7 (100)	2 (66.7)	12 (100)	12 (67)	
Present	7 (15.9)	0 (0)	0 (0)	1 (33.3)	0 (0)	6 (33)	
Capsular invasion^b^							
Absent	16 (84.2)	4 (100)	N/A	0 (0)	12 (100)	N/A	0.001
Present	3 (15.8)	0 (0)	N/A	3 (100)	0 (0)	N/A	
N/A	25	0	7	0	0	18	

*n* Number; *F* Female; *M* Male; *MUT* Mutant; *WT* Wild-type; *I* Isthmus; *L* Left; *MF* Multifocal; *R* Right; *N/A* Not assessed; *FA* Follicular adenoma; *I-FVPTC* Infiltrative follicular variant papillary thyroid carcinoma; *iE-FVPTC* invasive Encapsulated follicular variant papillary thyroid carcinoma; *NIFTP* Noninvasive follicular thyroid neoplasm with papillary-like nuclear features; *PTC* Papillary thyroid carcinoma; *IQR* Interquartile range

^a^Median (IQR)

^b^n (%)

^c^Kruskal-Wallis rank sum test; Fisher’s exact test

### Combined LDA and MALDI approach for the classification of thyroid nodules

**Fig. 1:**
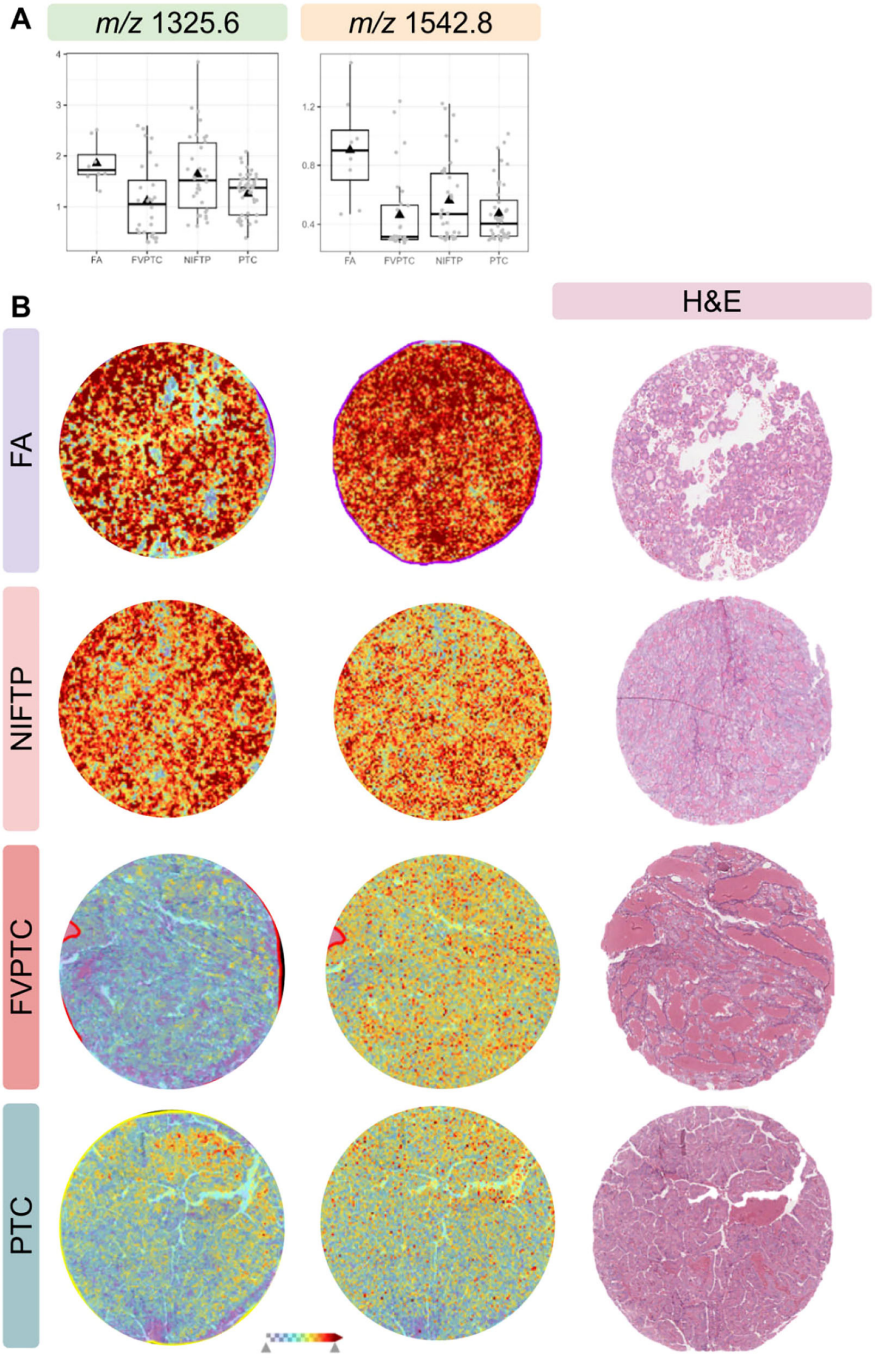
**A** Molecular distribution of the H4_HUMAN (*m/z* 1325.6) and DDX42 (*m/z* 1542.8) signals by category of thyroid lesions. The black triangle represents the mean, while the dots represent the single observations. **B** MALDI-MSI images showing the spatial localization of the signals. FA Follicular adenoma, FVPTC Follicular variant papillary thyroid carcinoma, NIFTP Noninvasive follicular thyroid neoplasm with papillary-like nuclear features, PTC Papillary thyroid carcinoma

After appropriate filtering and selection of MALDI-MSI features from the original 373 *m/z* signals, the LDA identified nine relevant molecular markers achieving a balanced accuracy of 73% in distinguishing NIFTPs from the other thyroid lesions. The model also performed well in identifying the FA and PTC classes (Sn = 75%), showing the highest PPV (87%) in the PTC class, followed by the FA and NIFTP categories (PPV = 67% and 62%, respectively) (Table 2A). The distribution of the nine LDA-selected features among the different tumor classes revealed interesting relationships of NIFTPs with both malignant (e.g., *m/z* 1542.777 and 1586.813 signals in FVPTC/PCT) and benign (e.g., *m/z* 1094.621 and 1325.645 signals in FA) lesions in terms of protein expression patterns, highlighting the controversial biological behavior of this neoplasm (Fig. 1 and Table S1).

**Table 2:** Performance and predicted classes of the LDA models developed to discriminate among the different lesions (**A**) and according to NRAS mutational status (**B**)

A										
Class	n	Predicted as FA	Predicted as FVPTC	Predicted as NIFTP	Predicted as PTC	Sn	Sp	PPV	NPV	Balanced Accuracy
FA	8	6	0	2	0	0.75	0.97	0.67	0.98	0.86
FVPTC	28	1	18	7	2	0.64	0.83	0.55	0.88	0.73
NIFTP	34	2	8	21	3	0.62	0.84	0.62	0.84	0.73
PTC	44	0	7	4	33	0.75	0.93	0.87	0.86	0.84

B										
Class	n	TN	FP	TP	FN	Sn	Sp	PPV	NPV	Balanced Accuracy
WT vs. MUT	62 (32 vs. 30)	23	9	29	1	0.97	0.72	0.76	0.96	0.84

*n* Number; *FA* Follicular adenoma; *FVPTC* Follicular variant papillary thyroid carcinoma; *NIFTP* Noninvasive follicular thyroid neoplasm with papillary like-nuclear features; *PTC* Papillary thyroid carcinoma; *Sn* Sensitivity; *Sp* Specificity; *PPV* Positive predictive value; *NPV* Negative predictive value; *WT* Wild-type; *MUT* Mutant; *TN* True negatives; *FP* False positives; *TP* True positives; *FN* False negatives

### Combined LDA and MALDI approach to discriminate between mNRAS and wtNRAS cases

**Fig. 2:**
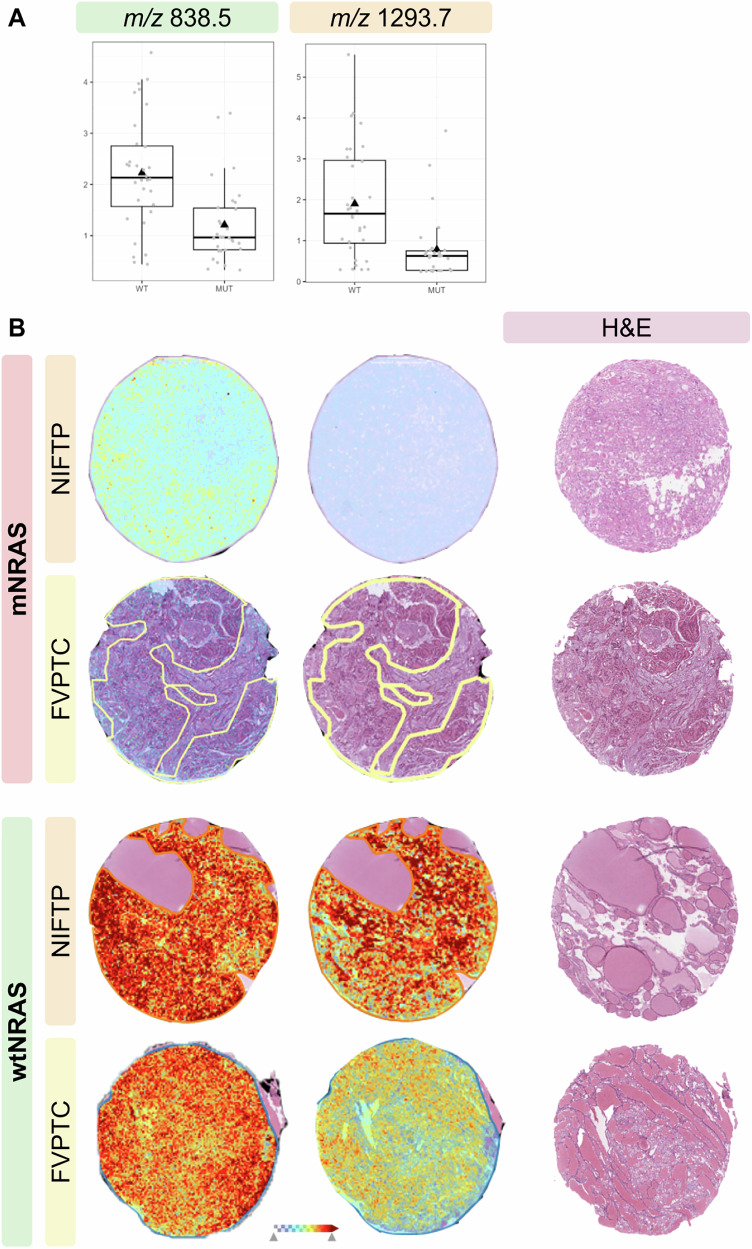
**A** Molecular distribution of the CO4B (*m/z* 838.5) and PDIA1 (*m/z* 1293.7) signals by NRAS mutational status. The black triangle represents the mean, while the dots represent the single observations. **B** MALDI-MSI images showing the spatial localization of the signals. FVPTC Follicular variant papillary thyroid carcinoma, NIFTP Noninvasive follicular thyroid neoplasm with papillary-like nuclear features, mNRAS NRAS mutant, wtNRAS NRAS-wild-type

Comparing mNRAS and wtNRAS cases, the LDA allowed a significant reduction of relevant signals, selecting 167 and 52 *m/z* peaks for FVPTC and NIFTP, respectively. 51 of these signals were overexpressed in wtNRAS compared to mNRAS cases, regardless of histology (Table S2). Based on the 19 most relevant molecular markers (Table S2), the model achieved a balanced accuracy of 84%, correctly classifying 29 out of 30 mNRAS ROIs and 23 out of 32 wtNRAS cases, with a Sn of 97% and a Sp of 72% (Table 2B). The visual inspection of the distribution of some of these signals highlighted clear differences between mNRAS and wtNRAS cases, independently of histology (e.g., *m/z* 838.5 and 1293.7 signals, both overexpressed in wtNRAS cases. Figure 2).

### Signal identification through nLC–ESI-MS/MS

By means of nLC–ESI-MS/MS, nine of the proteins with different expression patterns among the various classes of thyroid neoplasms were putatively identified. Among them, ATP-dependent RNA helicase DDX42 (*m/z* 1542.777, DDX42) showed a similar distribution between NIFTPs and FVPTCs/PTCs, while the distribution of the Histone H4 signal (*m/z* 1325.645, H4) was similar between NIFTPs and FAs. In addition, Complement C4-B (*m/z* 838.5, CO4B) and Protein disulfide-isomerase, also known as cellular thyroid hormone-binding protein (*m/z* 1293.7, PDIA1), were both overexpressed in wtNRAS cases independently of histology (a complete list of the identified proteins is reported in Table S3). These proteins are involved in the organization of chromatin structure (H4) and regulation of mRNA splicing (DDX42), as well as in the degradation of misfolded proteins and modulation of thyroid hormone receptor function (PDIA1) [16–18], suggesting that a disruption in these key cellular processes might play a role in the pathogenesis of the lesions studied.

## Discussion

Extensive research efforts have focused on identifying novel biomarkers for challenging thyroid lesions, with the aim to improve diagnostic accuracy and define useful prognostic indicators to guide patient management [19]. The introduction of innovative molecular markers able to fill the existing accuracy gap of the combined ultrasound-FNAC approach would more precisely identify patients who are truly candidates for invasive therapeutic procedures. Follicular-patterned thyroid lesions represent a particularly challenging group of neoplasms, encompassing entities with distinct behaviors and different malignant potential. Among these, NIFTPs have been recently “downgraded” as indolent neoplasms [20], although their biology is still far from being fully understood. Moreover, the presence of “atypical” cases, often bearing RAS mutations, further complicates efforts to distinguish NIFTPs from the other follicular-patterned lesions [21].

MALDI-MSI has emerged as a promising tool for biomarker discovery, enabling the identification of the specific molecular signals associated with the analyzed lesions [22]. Given the considerable amount of information deriving from MALDI-MSI analysis, we built an LDA-supervised approach for feature selection and classification based on proteomic data obtained from a group of challenging thyroid lesions (i.e., FAs, NIFTPs, FVPTCs, and PTCs). Among these, FAs were significantly larger at diagnosis compared to the other neoplasms, reflecting their indolent course and the subsequent late diagnosis. As expected, NRAS mutations were significantly more frequent in follicular-patterned lesions (i.e., NIFTP and FVPTC) compared to classic PTCs. Our approach enabled the identification of specific molecular markers that correspond to follicular-patterned lesions, including NIFTPs and FVPTCs, while also providing valuable insights into the complex behavioral trajectories of NIFTPs with respect to morphologically similar neoplasms. This, in turn, could be useful for discerning whether such behavior more closely resembles that of the malignant class (PTC, FVPTC) as opposed to the benign category (FA). Specifically, the similar downregulation of DDX42 in NIFTPs and FVPTCs/PTCs compared to FAs may suggest that the former lesions share similar alterations in pre-mRNA processing. Splicing abnormalities could thus represent important mechanisms underlying the development and progression of these neoplasms, similar to what has been observed in other tumor types [23]. On the other hand, the comparable distribution of the Histone H4 signal between NIFTPs and FAs and its underexpression in FVPTCs/PTCs may indicate a higher burden of chromatin structural aberrations in the latter category of lesions, which is a hallmark of a malignant behavior. Indeed, downregulation of conventional histone proteins may reflect the increased expression of modified histone variants, which have been identified as key contributors to tumor progression and invasiveness in several malignancies, including breast and colorectal cancer [24].

Apart from their pathobiological significance, the identified signals may be valuable for differentiating between these challenging thyroid lesions in routine pathological practice. Indeed, previous work by our group has shown a significant downregulation of the DDX42 and Histone H4 proteins in RAS-mutant compared to RAS-wild type NIFTPs from a separate cohort of patients, encouraging further validation of these markers as ancillary tools to assist in the differential diagnosis of borderline thyroid neoplasms [25]. This would require assessing the expression of the identified markers through immunohistochemistry in a separate and, possibly, larger sample of FAs, PTCs, FVPTCs, and NIFTPs. Specifically, the commercial availability of antibodies for the immunoenzymatic detection of the DDX42 and Histone H4 proteins may warrant the recruitment of additional subjects to establish a validation set of sufficient size for statistically robust analyses.

Finally, the comparison of wtNRAS vs. mNRAS cases among follicular-patterned lesions (i.e., FVPTC and NIFTP) enabled the identification of molecular signals associated with NRAS Q61R positivity, potentially providing insights into the distinct pathogenic pathways through which these lesions develop. Among the differentially expressed signals, we were able to putatively identify the Complement C4-B and PDIA1 proteins, which we found to be overexpressed in wtNRAS compared to mNRAS cases. Altered expression of both of these proteins has already been implicated in the biology of thyroid neoplasms: PTC cells have been shown to escape host immunosurveillance through the synthesis of anti-idiotypic immunoglobulin G (IgG) molecules that bind to and neutralize host IgGs directed against tumor antigens. The subsequent formation of immune complexes leads to the activation of the classical complement pathway, explaining the observed co-localization of IgGs and complement proteins (including C4) in PTC tissues [26]. Similarly, members of the protein disulfide isomerase family, to which PDIA1 belongs, have been identified as pivotal factors in promoting the progression and dissemination of several cancer types, including PTC [27]. PDIs, which are redox proteins bearing thioredoxin-like domains [28], have pleiotropic roles in regulating thyroid hormone receptor signaling, misfolded protein degradation, and progression through the cell cycle [29]. Therefore, aberrant PDIA1 expression may contribute to tumor development and progression by disrupting intracellular protein homeostasis, as well as by altering the transcriptional responses elicited by the binding of thyroid hormones to their receptors. In addition, reduced expression of another member of the PDI family, namely PDIA3, has been found to correlate with lymph node metastasis and poor prognosis in PTC patients [30]. Overall, these observations support the hypothesis that mNRAS and wtNRAS lesions are intrinsically distinct entities that rely on different pathways for tumor growth and immune evasion. Whether these biological differences lead to a distinct clinical behavior of mNRAS compared to wtNRAS tumors, regardless of histology, is still uncertain, warranting prospective clinical studies with large patient cohorts.

The wealth of discriminative features identified in our study further confirms the remarkable potential of MALDI-MSI to streamline biomarker discovery in the field of thyroid pathology, particularly when machine learning algorithms are applied to support data analysis. However, the implementation of this tool into the routine diagnostic practice is currently hampered by its relatively high costs, limited availability, and technical constraints regarding sample preparation for proteomic analyses. Thus, future advancements to improve the accessibility and scalability of this methodology are highly anticipated. This may be achieved by defining a restricted panel of relevant markers and testing their expression through multiplex spatial imaging approaches, such as MALDI-HiPLEX-IHC, which allows to precisely map the distribution of proteins of interest in FFPE tissues [31]. As shown by our group using clear cell renal cell carcinoma models, the integration of MALDI-HiPLEX-IHC with untargeted spatial proteomics enables the spatial localization of antigens of interest as well as the characterization of the tumor immune microenvironment, requiring only a single FFPE tissue section [32]. Based on these promising results, we are planning to extend the applicability of our workflow also to challenging thyroid lesions.

Although promising, our preliminary application of the LDA model to distinguish between histologically similar thyroid lesions has some limitations: due to the small number of subjects enrolled in our study, we decided to analyze the full patient cohort without splitting our dataset, as this would have resulted in underpowered training folds. For similar reasons, our proteomic analyses were performed considering I-FVPTCs and iE-FVPTCs as a single neoplastic category (i.e., FVPTC). Thus, the full generalizability of our model still requires further validation with external cohorts.

Moreover, the main focus of our study was to investigate putative biomarkers to facilitate the differential diagnostic process of borderline thyroid lesions. Whether our methodology can also identify markers correlated with relevant histopathological features (e.g., the presence of capsular and/or lymphovascular invasion) remains to be explored. Nevertheless, since the identified proteins may aid in the distinction between NIFTPs and histologically similar invasive neoplasms, they might serve as indirect indicators of tumor invasiveness irrespective of histology. Again, these preliminary findings warrant further validation studies with larger patient cohorts.

Finally, our analyses were carried out on a relatively limited set of neoplasms, so additional efforts are needed to extend the applicability of our approach to other challenging entities.

## Conclusion

Biomarker discovery is crucial for diagnostic and prognostic purposes, particularly in the context of thyroid lesions. Our work showed promise in identifying characteristic protein patterns associated with specific thyroid tumors, including NIFTPs, also considering their NRAS mutational status. The generalizability of our findings will require further validation on external cohorts and on a broader and more heterogeneous group of thyroid neoplasms.

## Supplementary information

Supplementary Material

## Data Availability

The data that support the findings of this study are not publicly available as they contain information that could compromise the privacy of research participants, but will be made available by the corresponding author (GC) upon reasonable request.
