# Supplementary material for: Biomarker identification through spatial proteomics for the characterization of indeterminate thyroid nodules
Source: Endocrine. 2025 Aug 11;90(2):800–9. doi: 10.1007/s12020-025-04383-9 (PMC12572012; doi:10.1007/s12020-025-04383-9)
Supplement: Supplementary file 1 — Supplementary Material [file 12020_2025_4383_MOESM1_ESM.docx]

**Supplementary material – Biomarker Identification through Spatial Proteomics for the Characterization of Indeterminate Thyroid Nodules – Endocrine – Authors:** Giulia Capitoli, Antonio Maria Alviano, Nicole Monza, Lisa Pagani, Isabella Piga, Davide Paolo Bernasconi, Angela Greco, Davide Leni, Alice Maggioni, Andrea-Valer Gatti, Fausto Maffini, Nicola Fusco, Mattia Garancini, Fulvio Magni, Stefania Galimberti, Fabio Pagni, Vincenzo L’Imperio, Vanna Denti – **Corresponding Author:** Giulia Capitoli, PhD, Assistant Professor of Biostatistics, Bicocca Bioinformatics Biostatistics and Bioimaging B4 Research Centre, Department of Medicine and Surgery, University of Milano-Bicocca, Via Cadore 48, 20900, Monza, Italy. E-mail: [giulia.capitoli@unimib.it](mailto:giulia.capitoli@unimib.it). Tel: +39 0264488333.

# **Table S1** Intensity distribution of the relevant *m/z* signals selected by the LDA model to discriminate among the various lesions

| ***m/z* signal** | **FA**, n = 8*^a^* | **FVPTC**, n = 28*^a^* | **NIFTP**, n = 34*^a^* | **PTC**, n = 44*^a^* | **p-value***^b^* | **q-value***^c^* |
| --- | --- | --- | --- | --- | --- | --- |
| 1094.621 | 1.57 (1.35, 1.86) | 0.96 (0.60, 1.25) | 1.21 (0.86, 1.64) | 0.99 (0.77, 1.20) | <0.001 | <0.001 |
| 1849.906 | 0.79 (0.69, 0.97) | 0.64 (0.47, 0.69) | 0.78 (0.74, 0.96) | 0.69 (0.60, 0.82) | <0.001 | <0.001 |
| 1325.645 | 1.72 (1.64, 2.03) | 1.05 (0.49, 1.52) | 1.52 (0.98, 2.25) | 1.37 (0.84, 1.54) | 0.001 | 0.001 |
| 1542.777 | 0.90 (0.70, 1.04) | 0.31 (0.30, 0.53) | 0.47 (0.32, 0.75) | 0.40 (0.32, 0.56) | <0.001 | <0.001 |
| 1511.755 | 0.99 (0.86, 1.14) | 0.51 (0.38, 0.73) | 0.84 (0.51, 1.17) | 0.67 (0.50, 0.98) | <0.001 | <0.001 |
| 1625.877 | 0.51 (0.46, 0.56) | 0.35 (0.32, 0.42) | 0.42 (0.33, 0.50) | 0.53 (0.39, 0.68) | <0.001 | <0.001 |
| 1586.813 | 0.87 (0.78, 1.37) | 1.09 (0.88, 1.78) | 1.51 (1.30, 2.00) | 1.73 (1.44, 1.93) | 0.001 | 0.001 |
| 1198.728 | 2.12 (1.61, 2.61) | 0.84 (0.36, 1.50) | 1.62 (0.79, 2.51) | 2.02 (1.21, 3.08) | <0.001 | <0.001 |
| 1199.752 | 1.51 (1.11, 1.94) | 0.60 (0.35, 1.11) | 1.18 (0.66, 1.83) | 1.43 (0.89, 2.22) | <0.001 | <0.001 |

^a^Median (IQR)

^b^Kruskal-Wallis rank sum test

^c^False discovery rate correction for multiple testing

FA, Follicular adenoma; n, Number; FVPTC, Follicular variant papillary thyroid carcinoma; NIFTP, Noninvasive follicular thyroid neoplasm with papillary-like nuclear features; PTC, Papillary thyroid carcinoma; IQR, Interquartile range

# **Table S2** Intensity distribution of the 51 *m/z* signals discriminating between mNRAS and wtNRAS cases within the FVPTC and NIFTP classes

| ***m/z* signal** | **FVPTC-MUT**, n = 10*^a^* | **FVPTC-WT**, n = 18*^a^* | **NIFTP-MUT**, n = 20*^a^* | **NIFTP-WT**, n = 14*^a^* | **p-value***^b^* | **q-value***^c^* |
| --- | --- | --- | --- | --- | --- | --- |
| 730.395^d^ | 0.31 (0.27, 0.44) | 0.99 (0.73, 1.43) | 0.68 (0.50, 1.05) | 1.22 (1.01, 1.47) | <0.001 | <0.001 |
| 731.418 | 0.24 (0.23, 0.32) | 0.89 (0.50, 1.14) | 0.58 (0.44, 0.81) | 1.27 (1.15, 1.73) | <0.001 | <0.001 |
| 746.381 | 0.20 (0.19, 0.27) | 0.71 (0.38, 1.03) | 0.45 (0.41, 0.62) | 1.30 (1.11, 2.17) | <0.001 | <0.001 |
| 775.527^d^ | 0.26 (0.24, 0.28) | 1.22 (0.83, 2.03) | 0.87 (0.75, 1.06) | 1.86 (1.62, 2.60) | <0.001 | <0.001 |
| 776.502 | 0.22 (0.21, 0.26) | 0.70 (0.50, 0.95) | 0.51 (0.48, 0.67) | 0.97 (0.82, 1.29) | <0.001 | <0.001 |
| 781.424 | 0.23 (0.22, 0.34) | 0.81 (0.45, 1.05) | 0.57 (0.45, 0.77) | 1.21 (1.15, 1.63) | <0.001 | <0.001 |
| 805.501^d^ | 0.26 (0.24, 0.38) | 0.67 (0.40, 0.98) | 0.55 (0.47, 0.75) | 1.01 (0.86, 1.47) | <0.001 | <0.001 |
| 824.485^d^ | 0.22 (0.22, 0.27) | 0.91 (0.48, 1.13) | 0.50 (0.45, 0.73) | 1.16 (1.12, 1.87) | <0.001 | <0.001 |
| 838.473^d^ | 0.62 (0.43, 0.77) | 1.87 (1.27, 2.31) | 1.26 (0.95, 1.71) | 2.57 (2.15, 3.46) | <0.001 | <0.001 |
| 842.543 | 0.35 (0.26, 0.48) | 0.80 (0.47, 1.10) | 0.65 (0.53, 1.11) | 1.28 (0.91, 1.82) | <0.001 | <0.001 |
| 860.479 | 0.29 (0.28, 0.36) | 0.89 (0.47, 1.03) | 0.53 (0.39, 0.84) | 1.14 (0.84, 1.60) | <0.001 | <0.001 |
| 908.463 | 0.37 (0.30, 0.52) | 1.13 (0.79, 1.44) | 0.86 (0.64, 1.24) | 1.62 (1.35, 1.86) | <0.001 | <0.001 |
| 934.538 | 0.24 (0.23, 0.26) | 0.78 (0.46, 1.06) | 0.53 (0.44, 0.69) | 1.18 (1.07, 1.54) | <0.001 | <0.001 |
| 936.536^d^ | 0.24 (0.23, 0.28) | 1.11 (0.55, 1.46) | 0.56 (0.48, 0.93) | 1.59 (1.37, 2.36) | <0.001 | <0.001 |
| 937.511^d^ | 0.23 (0.23, 0.25) | 0.93 (0.58, 1.56) | 0.56 (0.52, 0.83) | 1.74 (1.21, 2.74) | <0.001 | <0.001 |
| 980.548 | 0.24 (0.23, 0.25) | 0.47 (0.32, 0.59) | 0.39 (0.32, 0.54) | 0.88 (0.80, 1.14) | <0.001 | <0.001 |
| 1018.540 | 0.23 (0.23, 0.24) | 0.60 (0.38, 0.84) | 0.47 (0.42, 0.66) | 1.04 (0.89, 1.82) | <0.001 | <0.001 |
| 1075.613^d^ | 0.26 (0.25, 0.28) | 1.59 (0.89, 3.60) | 1.04 (0.91, 1.38) | 3.41 (2.81, 3.91) | <0.001 | <0.001 |
| 1097.595 | 0.51 (0.38, 0.57) | 1.05 (0.87, 1.67) | 0.91 (0.77, 1.21) | 1.63 (1.43, 1.90) | <0.001 | <0.001 |
| 1104.613 | 0.24 (0.24, 0.25) | 0.72 (0.47, 1.21) | 0.58 (0.52, 0.76) | 1.39 (1.21, 1.74) | <0.001 | <0.001 |
| 1110.584 | 0.35 (0.29, 0.37) | 1.23 (0.87, 2.06) | 0.99 (0.94, 1.15) | 2.08 (1.69, 2.39) | <0.001 | <0.001 |
| 1111.607 | 0.90 (0.64, 1.24) | 2.04 (1.66, 2.70) | 2.02 (1.55, 2.60) | 2.99 (2.41, 3.71) | <0.001 | <0.001 |
| 1140.607^d^ | 0.32 (0.30, 0.40) | 1.15 (0.80, 1.64) | 0.90 (0.65, 1.24) | 1.62 (1.36, 2.18) | <0.001 | <0.001 |
| 1271.739^d^ | 0.3 (0.3, 0.3) | 3.1 (1.5, 5.8) | 2.0 (1.6, 2.3) | 5.6 (4.6, 7.8) | <0.001 | <0.001 |
| 1288.749 | 0.26 (0.26, 0.28) | 0.62 (0.48, 1.04) | 0.50 (0.45, 0.69) | 1.10 (0.78, 1.21) | <0.001 | <0.001 |
| 1292.697 | 0.27 (0.26, 0.28) | 0.45 (0.34, 0.61) | 0.39 (0.34, 0.63) | 0.83 (0.63, 1.06) | <0.001 | <0.001 |
| 1293.720^d^ | 0.27 (0.26, 0.27) | 1.19 (0.59, 1.97) | 0.71 (0.63, 0.88) | 1.97 (1.41, 3.24) | <0.001 | <0.001 |
| 1306.685 | 0.29 (0.29, 0.31) | 0.49 (0.41, 0.69) | 0.47 (0.41, 0.64) | 0.92 (0.68, 1.16) | <0.001 | <0.001 |
| 1315.702 | 0.26 (0.26, 0.27) | 0.83 (0.43, 1.06) | 0.47 (0.43, 0.74) | 1.10 (0.86, 1.74) | <0.001 | <0.001 |
| 1316.725 | 0.26 (0.24, 0.26) | 0.67 (0.36, 0.88) | 0.39 (0.37, 0.64) | 0.86 (0.69, 1.32) | <0.001 | <0.001 |
| 1323.671 | 0.31 (0.28, 0.35) | 0.78 (0.46, 1.13) | 0.57 (0.47, 0.98) | 1.21 (0.86, 1.64) | <0.001 | <0.001 |
| 1337.707 | 0.29 (0.28, 0.31) | 0.90 (0.51, 1.16) | 0.50 (0.42, 1.05) | 1.42 (1.01, 1.78) | <0.001 | <0.001 |
| 1361.687^d^ | 0.29 (0.28, 0.29) | 1.70 (1.04, 3.50) | 1.26 (1.06, 1.54) | 3.43 (2.91, 4.40) | <0.001 | <0.001 |
| 1380.793 | 0.27 (0.26, 0.27) | 0.70 (0.48, 1.25) | 0.56 (0.50, 0.67) | 1.41 (0.90, 2.25) | <0.001 | <0.001 |
| 1383.693^d^ | 0.28 (0.25, 0.28) | 0.87 (0.49, 1.31) | 0.60 (0.51, 0.70) | 1.32 (0.96, 2.02) | <0.001 | <0.001 |
| 1384.741^d^ | 0.28 (0.27, 0.28) | 1.01 (0.63, 1.73) | 0.77 (0.69, 1.06) | 2.05 (1.59, 2.47) | <0.001 | <0.001 |
| 1385.764^d^ | 0.28 (0.28, 0.29) | 0.95 (0.54, 1.41) | 0.74 (0.60, 0.85) | 1.69 (1.26, 2.03) | <0.001 | <0.001 |
| 1406.722^d^ | 0.30 (0.29, 0.32) | 1.31 (0.75, 2.05) | 0.82 (0.72, 1.06) | 2.94 (1.94, 3.39) | <0.001 | <0.001 |
| 1430.702 | 0.28 (0.28, 0.30) | 0.72 (0.45, 1.44) | 0.61 (0.50, 0.76) | 1.51 (1.13, 1.71) | <0.001 | <0.001 |
| 1448.784 | 0.29 (0.28, 0.31) | 0.59 (0.39, 0.86) | 0.44 (0.37, 0.61) | 0.97 (0.65, 1.40) | <0.001 | <0.001 |
| 1464.819 | 0.33 (0.31, 0.35) | 1.21 (0.86, 2.68) | 1.44 (0.94, 1.68) | 2.70 (2.08, 3.37) | <0.001 | <0.001 |
| 1465.721^d^ | 1.32 (0.82, 1.65) | 2.55 (1.68, 3.06) | 2.35 (1.83, 2.80) | 3.50 (2.73, 4.10) | <0.001 | <0.001 |
| 1486.800 | 0.29 (0.28, 0.30) | 0.65 (0.43, 1.05) | 0.56 (0.52, 0.65) | 1.19 (0.90, 1.46) | <0.001 | <0.001 |
| 1508.757 | 0.40 (0.34, 0.50) | 0.89 (0.70, 1.22) | 0.91 (0.62, 1.32) | 1.69 (1.14, 1.82) | <0.001 | <0.001 |
| 1825.951 | 0.36 (0.34, 0.38) | 0.64 (0.44, 0.98) | 0.50 (0.43, 0.56) | 0.84 (0.72, 1.24) | <0.001 | <0.001 |
| 1844.764 | 0.34 (0.34, 0.37) | 0.51 (0.40, 0.67) | 0.43 (0.41, 0.51) | 0.95 (0.78, 1.26) | <0.001 | <0.001 |
| 1861.798 | 0.37 (0.37, 0.39) | 0.94 (0.63, 1.51) | 0.61 (0.52, 0.85) | 2.18 (1.77, 2.90) | <0.001 | <0.001 |
| 1882.025 | 0.37 (0.36, 0.38) | 0.55 (0.48, 0.83) | 0.49 (0.44, 0.55) | 1.03 (0.71, 1.83) | <0.001 | <0.001 |
| 1932.056^d^ | 0.38 (0.36, 0.39) | 0.84 (0.62, 1.45) | 0.63 (0.60, 0.75) | 1.55 (1.29, 2.07) | <0.001 | <0.001 |
| 1954.037 | 0.37 (0.35, 0.37) | 0.64 (0.54, 1.14) | 0.65 (0.56, 0.72) | 1.10 (0.97, 1.73) | <0.001 | <0.001 |
| 2023.953^d^ | 0.36 (0.35, 0.37) | 0.50 (0.42, 0.73) | 0.42 (0.39, 0.49) | 0.90 (0.83, 1.08) | <0.001 | <0.001 |

# ^a^Median (IQR)

^b^Kruskal-Wallis rank sum test

^c^False discovery rate correction for multiple testing

^d^Subgroup of the 19 relevant features selected by the LDA model to discriminate between mNRAS and wtNRAS lesions

MUT, Mutant; n, Number; WT, Wild-type; FVPTC, Follicular variant papillary thyroid carcinoma; NIFTP, Noninvasive follicular thyroid neoplasm with papillary-like nuclear features; IQR, Interquartile range

#

#

# **Table S3** Putative identification of the relevant *m/z* signals selected by the LDA model to discriminate between the various lesions and according to NRAS mutational status

| **Putative assignments (unique)** | | | | | |
| --- | --- | --- | --- | --- | --- |
| ***m/z* signal** | **Protein accession** | **Peptide sequence** | ***m/z* nLC-MS/MS** | **Δppm** | **ΔDa** |
| 775.53^a^ | THYG_HUMAN | R.VLQFIR.E | 775.482 | 57 | 0.04 |
| 805.50^a^ | CO6A3_HUMAN | K.ALEFVAR.N | 805.454 | 59 | 0.05 |
| 824.49^b^ | IGHG2_HUMAN | K.GLPAPIEK.T | 824.486 | -1 | 0.00 |
| 838.47^a^ | CO4B_HUMAN | K.ASSFLGEK.A | 838.429 | 53 | 0.04 |
| 936.54^a^ | THYG_HUMAN | R.ATNSQLFR.R | 936.488 | 51 | 0.05 |
| 1075.61^a^ | THYG_HUMAN | K.GGFIPGSLTAR.S | 1075.588 | 23 | 0.02 |
| 1271.74^a^ | THYG_HUMAN | K.VIFDANAPVAVR.S | 1271.709 | 23 | 0.03 |
| 1293.72^b^ | PDIA1_HUMAN | K.MDSTANEVEAVK.V | 1293.615 | 81 | 0.10 |
| 1325.65^a,c^ | H4_HUMAN | R.DNIQGITKPAIR.R | 1325.750 | -79 | -0.11 |
| 1361.69^a^ | THYG_HUMAN | R.FLQGDHFGTSPR.T | 1361.656 | 23 | 0.03 |
| 1384.74^a^ | THYG_HUMAN | R.LAAQSTLSFYQR.R | 1384.717 | 18 | 0.02 |
| 1406.72^a,c^ | THYG_HUMAN | R.FSPDDSAGASALLR.S | 1406.689 | 24 | 0.03 |
| 1511.76^c^ | ALBU_HUMAN | K.VPQVSTPTLVEVSR.N | 1511.838 | -55 | -0.08 |
| 1542.78^a^ | DDX42_HUMAN | K.ALQEGAEIVVCTPGR.L | 1542.795 | -11 | -0.02 |
| 1625.88^b^ | PROF1_HUMAN | R.DSLLQDGEFSMDLR.T | 1625.735 | 87 | 0.14 |
| **Putative assignments (not unique)** | | | | | |
| ***m/z* signal** | **Protein accession** | **Peptide sequence** | ***m/z* nLC-MS/MS** | **Δppm** | **ΔDa** |
| 937.51 | PEBP1_HUMAN | K.LYEQLSGK | 937.497 | 15 | 0.01 |
|  | NUCL_HUMAN | K.TGISDVFAK.N | 937.496 | 17 | 0.02 |
| 1094.62 | RS16_HUMAN | K.LLEPVLLLGK.E | 1094.718 | -89 | -0.10 |
|  | CH10_HUMAN | R.DGDILGKYVD | 1094.523 | 89 | 0.10 |
| 1140.61 | RS5_HUMAN | R.QAVDVSPLRR.V | 1140.645 | -33 | -0.04 |
|  | FIBA_HUMAN | R.GSESGIFTNTK.E | 1140.550 | 50 | 0.06 |
|  | TR150_HUMAN | K.GSFSDTGLGDGK.M | 1140.516 | 80 | 0.09 |
| 1198.73 | PEPL_HUMAN | R.EAEVLLLQQR.V | 1198.675 | 44 | 0.05 |
|  | H14_HUMAN | K.ASGPPVSELITK.A | 1198.664 | 53 | 0.06 |
|  | SEPT9_HUMAN | K.QVENAGAIGPSR.F | 1198.611 | 98 | 0.12 |
| 1199.75 | HSP7C_HUMAN | K.DAGTIAGLNVLR.I | 1199.674 | 65 | 0.08 |
|  | TIF1B_HUMAN | K.ADVQSIIGLQR.F | 1199.646 | 88 | 0.11 |
| 1383.69 | AHNK_HUMAN | K.VDINAPDVEVQGK.V | 1383.703 | -7 | -0.01 |
|  | K2C1_HUMAN | K.SLNNQFASFIDK.V | 1383.687 | 4 | 0.01 |
|  | PEDF_HUMAN | K.LQSLFDSPDFSK.I | 1383.677 | 11 | 0.02 |
| 1385.76 | K2C7_HUMAN | R.AKQEELEAALQR.G | 1385.733 | 22 | 0.03 |
|  | ATPB_HUMAN | R.IMNVIGEPIDER.G | 1385.702 | 45 | 0.06 |
|  | NID2_HUMAN | R.ENLLEHYGGTPR.D | 1385.678 | 62 | 0.09 |
| 1465.72 | HS71A_HUMAN | K.AQIHDLVLVGGSTR.I | 1465.805 | -58 | -0.08 |
|  | SPTB2_HUMAN | R.LAEISDVWEEM(+15.99)K.T | 1465.681 | 27 | 0.04 |
| 1586.81 | CO6A3_HUMAN | R.LQPVLQPLPSPGVGGK.R | 1586.923 | -69 | -0.11 |
|  | BGAL_HUMAN | R.AYVAVDGIPQGVLER.N | 1586.840 | -17 | -0.03 |
|  | KCRB_HUMAN | K.LAVEALSSLDGDLAGR.Y | 1586.828 | -9 | -0.01 |
| 1849.91 | FREM2_HUMAN | R.KTDPPSADAGTDYVGISR.N | 1849.878 | 15 | 0.03 |
|  | HAP28_HUMAN | R.QYTSPEEIDAQLQAEK.Q | 1849.861 | 24 | 0.04 |

^a^Co-loc peptides or confirmed by in-situ MS/MS in other thyroid datasets

^b^Unique match with nLC-MS/MS

^c^Literature-confirmed

nLC-MS/MS, Nanoscale liquid chromatography tandem mass spectrometry; ppm, Parts per million; Da, Dalton


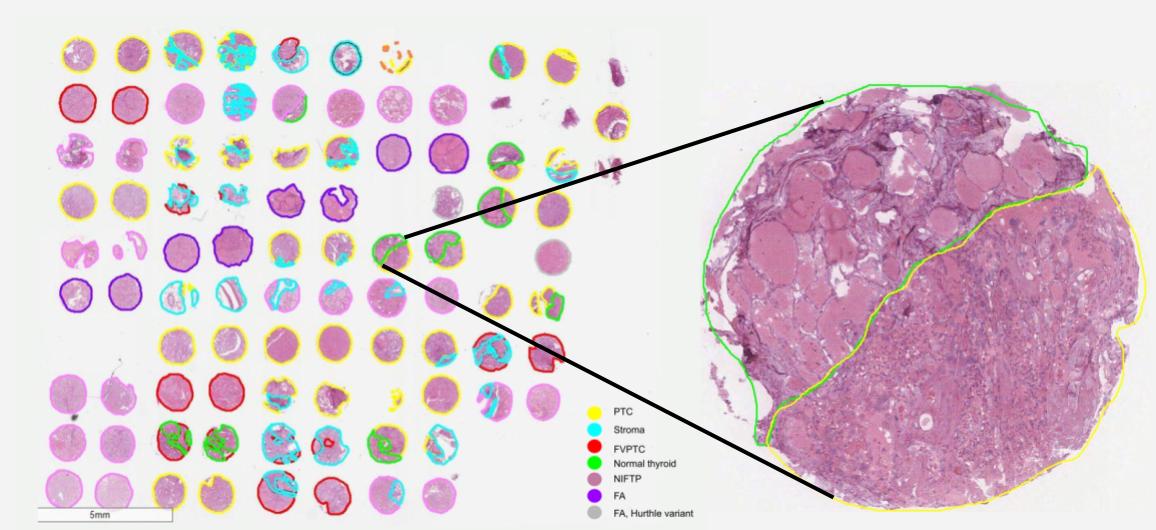


**Fig. S1** Cores from the original TMA annotated by the thyroid pathologist for the identification of stroma, non-neoplastic thyroid parenchyma, and tumor regions, with the various tumor histotypes labeled accordingly. A representative core with the corresponding annotations is shown at higher magnification in the inset. TMA, Tissue microarray; FA, Follicular adenoma; FVPTC, Follicular variant papillary thyroid carcinoma; NIFTP, Noninvasive follicular thyroid neoplasm with papillary-like nuclear features; PTC, Papillary thyroid carcinoma
